# Self-Phase-Matched Second-Harmonic and White-Light Generation in a Biaxial Zinc Tungstate Single Crystal

**DOI:** 10.1038/srep45247

**Published:** 2017-03-24

**Authors:** Pawel Osewski, Alessandro Belardini, Emilija Petronijevic, Marco Centini, Grigore Leahu, Ryszard Diduszko, Dorota A. Pawlak, Concita Sibilia

**Affiliations:** 1 Institute of Electronic Materials Technology, Wolczynska 133, 01-919, Warsaw, Poland; 2 University of Rome “La Sapienza”, 16 A. Scarpa, Rome, 00161, Italy; 3 Centre of New Technologies University of Warsaw, Banacha 2C, 02-097 Warsaw, Poland

## Abstract

Second-order nonlinear optical materials are used to generate new frequencies by exploiting second-harmonic generation (SHG), a phenomenon where a nonlinear material generates light at double the optical frequency of the input beam. Maximum SHG is achieved when the pump and the generated waves are in phase, for example through birefringence in uniaxial crystals. However, applying these materials usually requires a complicated cutting procedure to yield a crystal with a particular orientation. Here we demonstrate the first example of phase matching under the normal incidence of SHG in a biaxial monoclinic single crystal of zinc tungstate. The crystal was grown by the micro-pulling-down method with the (102) plane perpendicular to the growth direction. Additionally, at the same time white light was generated as a result of stimulated Raman scattering and multiphoton luminescence induced by higher-order effects such as three-photon luminescence enhanced by cascaded third-harmonic generation. The annealed crystal offers SHG intensities approximately four times larger than the as grown one; optimized growth and annealing conditions may lead to much higher SHG intensities.

Crystalline materials lacking inversion symmetry can exhibit second-order nonlinear optical properties, which can give rise to the phenomenon of frequency doubling^1^, where an input (pump) wave generates a wave with twice the optical frequency in the medium. This process is called SHG and is used for nonlinear frequency conversion of laser pulses, intracavity and resonant frequency conversion, as well as frequency doubling in waveguides. For example, a green light laser (532 nm, Vis) is obtained by the frequency doubling of a Nd:YAG laser (1064 nm, NIR).

To achieve high-efficiency SHG, nonlinear optical materials need to satisfy three criteria: (i) be non-centrosymmetric, (ii) fulfill the phase-matching (PM) condition, and (iii) possess large nonlinear optical coefficients^2^, as illustrated in Fig. 1. The material must be non-centrosymmetric because the second order nonlinear electrical polarization term *P*^(2)^ = *ε*_*0*_*χ*^(2)^*EE* (where *ε*_*0*_ - vacuum permittivity, *χ*^(2)^ - second-order nonlinear optical susceptibility, *E -* electric field) in bulk systems is present only when the material lacks inversion symmetry. However, this criterion can be overcome by breaking the original crystal symmetry by stress^3^, edge and surface defects and inhomogeneities^4^, or multipolar contributions^5^.

Fulfilling the PM condition causes generated wave-amplitude contributions from different locations of the material to sum up in the phase domain. A variety of PM techniques have been developed, such as temperature and angular tuning of birefringent materials, quasi-phase matching or harnessing the photovoltaic effect^6^. Many natural crystals have nonlinear properties with large nonlinear coefficients for certain propagation and polarization field directions. However, because of the natural dispersion of the materials, PM cannot always be achieved. Usually, uniaxial materials (such as β-BaB_2_O_4_ (BBO), KH_2_PO_4_ (KDP)) are used to obtain PM and efficient SHG, whereas biaxial crystals are less commonly employed^7^.

Studying a composite material made of ZnO and ZnWO_4_ phases we observed unusual nonlinear properties, mostly in the latter phase. This led us to studying in detail second-harmonic-generation in the single crystal of zinc tungstate, looking for new efficient SHG material operating without complicated post growth procedure The biaxial crystal investigated here, zinc tungstate (ZnWO_4_), belongs to a group of wolframite-type tungstates with the general formula AWO_4_, where A = Mg, Mn, Fe, Co, Ni, Zn or Cd. Zinc tungstate is a technologically important material that has been used in X-ray and gamma-scintillators^8^, solid state laser hosts^9^, optical^10^ and acoustic fibers^11^, sensors^12^ and phase-change optical recording media^13^. Recently, new applications for this material have emerged, such as rare-event detectors^14^, for example, for the detection of non-baryonic dark matter, 0v double-beta decay and radioactive decay of very long-living isotopes.

The optical and nonlinear properties of AWO_4_ materials have been studied intensively^15^,^16^, as have the spectroscopic, laser and cubic nonlinear properties of ZnWO_4_ crystals^17^. Until now second-harmonic generation in ZnWO4 has not been reported, likely because it crystallizes in a monoclinic, centrosymmetric configuration, which results in a zero bulk, second-order nonlinearity.

Bulk ZnWO_4_ single crystals have previously been prepared only by the Czochralski method^18^ and the growth of high-quality crystals is difficult to achieve. This is due to the high partial pressure of WO_3_ at higher temperatures^19^, the deviation from stoichiometry^18^ and a non-homogeneous material formation during the solid state reaction. We show that, as a result of these defects, the symmetry of the crystal can be locally distorted, which together with multipolar polarization sources^20^ can provide a relatively strong second-order, nonlinear response.

Here, we demonstrate phase-matched second-harmonic generation and white-light generation at normal incidence in a biaxial monoclinic ZnWO_4_ single crystal grown by the micro-pulling-down method. To our knowledge, this is the first demonstration of efficient SHG in zinc tungstate. The applied growth method gives rise to single crystals with second-order nonlinearity and phase matching at normal incidence for light conversion 800–400 nm. Moreover, we avoided the use of expensive and sophisticated preparation techniques, such as special cutting procedures, that are typically used to produce nonlinear crystals. To understand the origin of the observed nonlinear effects, we present detailed studies of the generated second-harmonic and white light, including angular and spectral analysis, polarization dependence and modeling. Finally, we report on the possibility of further improving SHG efficiency by crystal annealing.

## Results and Discussion

A single-crystal rod of ZnWO_4_ was grown by the micro-pulling-down method (μ-PD)^21^, which enables fast growth of good quality crystals, and requires only a small amount of raw materials. The μ-PD was invented for the growth of single crystal fibers^22^,^23^; however, it has also been used for the growth/solidification of eutectic-based, photonic crystal-like materials^24^,^25^, metamaterials^26^ and bulk nanoplasmonic materials obtained by direct doping of glass matrices with plasmonic nanoparticles^27^.

**Figure 2:**
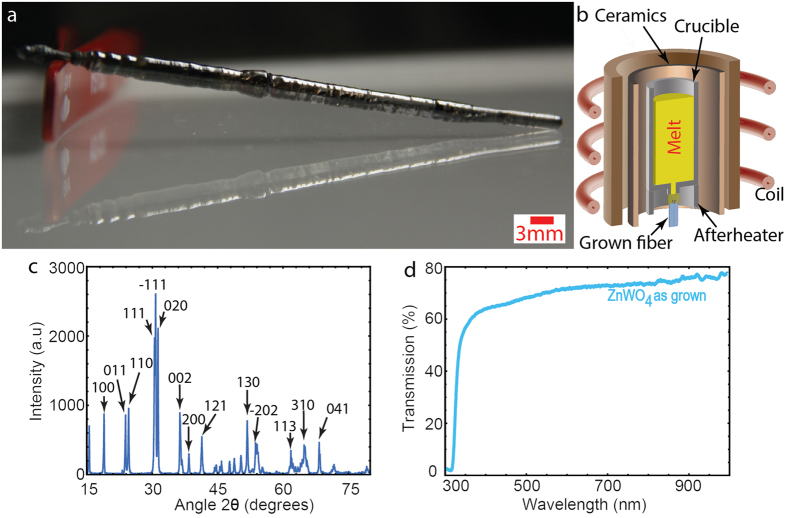
Single crystal ZnWO_4_ grown by the μ-PD method. (**a**) Image of the crystal. (**b**) Scheme of the micro-pulling-down method. (**c**) XRD spectrum confirming the single phase and providing the crystal orientation. (**d**) Transmission spectrum of the 300-μm-thick as-grown sample.

The obtained crystal was oriented with the (102) plane perpendicular to the growth direction, as confirmed by powder and single crystal X-ray Diffraction (XRD) shown in Fig. 2. The ZnWO_4_ crystallizes in a centrosymmetric, monoclinic system with space group P2/c. The unit cell parameters (a = 4.692 Å, b = 5.715 Å, c = 4.929 Å and β = 90.58°) are similar to the values presented previously (a = 4.690 Å, b = 5.7159 Å, c = 4.927 Å and β = 90.63°) for a crystal grown by the Czochralski method^28^. The samples were transparent starting from 325 nm and a direct bandgap of E_g_ = 3.87 eV was confirmed from the transmission spectra, as shown in Fig. 2d. The cut sample plane is tilted by 1.9° with respect to the <010> axis, as confirmed by XRD.

**Figure 3:**
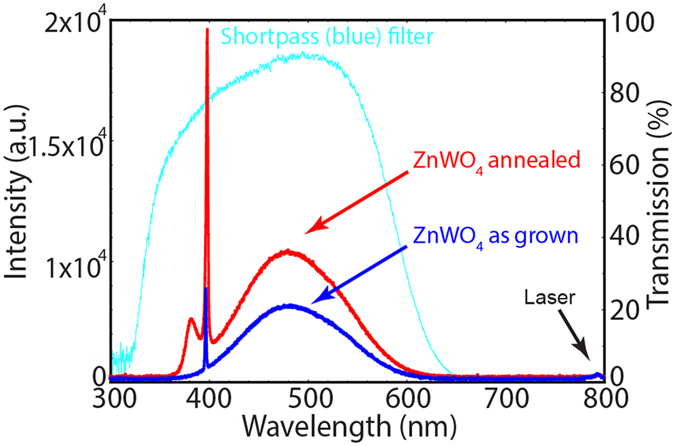
SH and white-light generation in ZnWO_4_ obtained when pumped with a 795-nm pulsed laser. Spectral analysis of the SH and white light generated by the as-grown (blue) and annealed (red) samples. The spectral character of the shortpass (blue) filter used is also shown (light blue).

Despite the centrosymmetric character of the crystal lattice, the ZnWO_4_ single crystal generated a sharp second-harmonic peak at 397 nm when pumped with 795 nm (~130 fs, 70 mW, 1 kHz) pulsed laser radiation. This can be observed in a spectral analysis of the nonlinear effects, in which the light transmitted through the sample at normal incidence was collected by an optical fiber and transmitted to a spectrometer (Fig. 3). To further increase the generation efficiency, the sample was annealed in air at 1000 °C for 12 h, which increased the SHG intensity by four times compared to the as-grown sample, also shown in Fig. 3.

**Figure 4:**
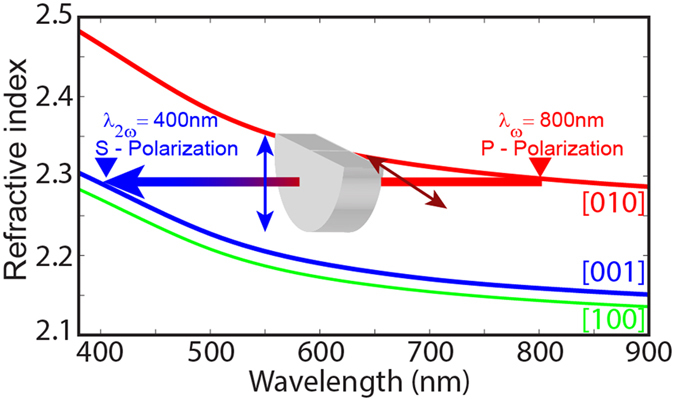
Self-phase matching in a ZnWO_4_ single crystal enabled by the biaxial character of the material. Dielectric function of ZnWO_4_ demonstrating two polarization-dependent and one polarization-independent dielectric functions^29^.

The ZnWO_4_ crystals are biaxial, with two similar principal dielectric functions for one optical axis and a third, significantly larger function for the second optical axis^29^. This enables phase matching of the 795-nm pumping beam with the ~400 nm second-harmonic-generated beam, as illustrated in Fig. 4. The as-grown crystal with the <010> axis in the plane of the sample cut at 90° is optimal for phase matching at normal incidence for a pump field polarized along the principal dielectric axis corresponding to the larger value of refractive index. Thus no additional special cutting of the crystal was necessary for fulfilling phase matching conditions.

**Figure 5:**
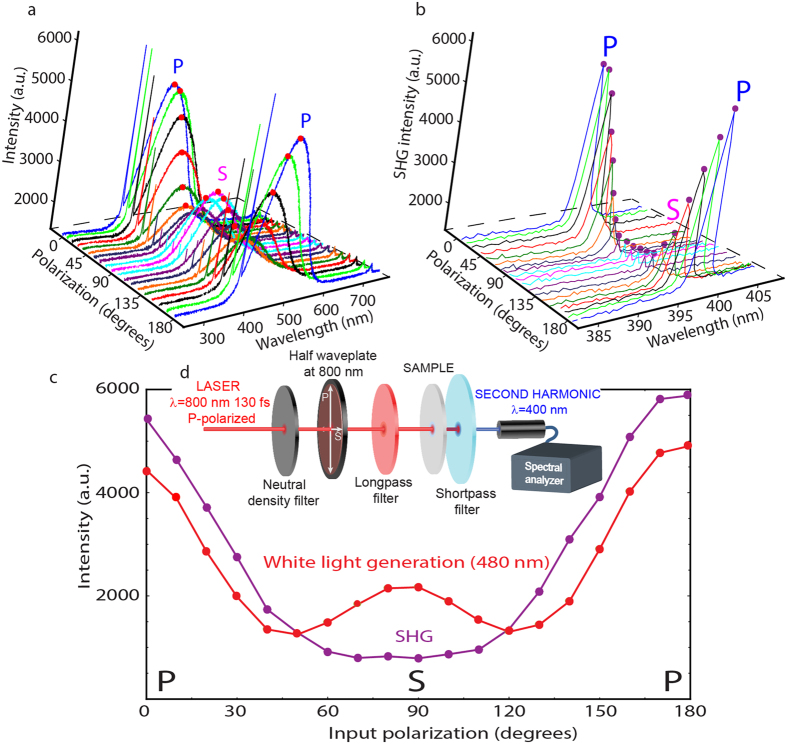
Polarization dependence of the nonlinear effects (SHG and white-light generation) in ZnWO_4_ single crystals. (**a**) Three-dimensional plots of the SHG and white light intensity as a function of the polarization of the output beam. (**b**) Spectrum and polarization dependence of SHG. (**c**) Polarization dependence of SHG and white-light generation. The red and violet points in (**c**) correspond to the red and violet points in (**a** and **b**), respectively. (**d**) Experimental setup used for the spectral analysis of the nonlinear effects.

In addition to second-harmonic generation, we observed broad white-light generation at 380–600 nm in our ZnWO_4_ crystalline samples, as shown in Fig. 3. The annealed samples exhibited an additional peak with an intensity maximum at 381 nm. The spectral characteristics of the generated white-light in both samples are different and spectral widths are smaller than that of the shortpass (blue) filter used to remove the traces of the pump laser beam, also shown in Fig. 3 (the measurement configuration is shown in Fig. 5d).

A variety of processes can lead to the generation of white light; these include three-photon luminescence and sum-frequency generation between the pump and stimulated Raman emission. Three-photon luminescence is enhanced by cascaded third-harmonic generation, where the pump frequency and the second-harmonic signal interact to induce a third-harmonic signal that excites high energy levels. These possible contributions are discussed and evaluated later and in the Supporting Material.

To obtain more information on the relation between the SHG and white-light generation, we performed angle- and polarization-dependent studies of the nonlinear effects (Fig. 5). The dependence of SHG on light polarization was analyzed by capturing the electromagnetic radiation generated by a sample, as a result of laser excitation, with an optical fiber and then transmitting it to a spectrometer equipped with a CCD matrix (Fig. 5a). The laser beam was initially 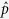-polarized and the polarization was changed during measurements by five degree steps towards 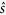 polarization. Employing a spectrometer for measurements allowed to record spectra of all nonlinear effects generated in the samples and enabled the differentiation of the SHG signal from the broad peak of white-light generation (Fig. 5b).

From the polarization and wavelength-dependent nonlinear signals we obtained, we found that the highest SHG intensity, (Fig. 5c,d), was obtained for excitation with a 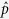-polarized beam, whereas for excitation with an 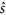-polarized beam, SHG was close to zero. This result confirms that the largest principal dielectric axis of the investigated ZnWO_4_ sample is aligned with the 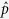 direction of the incident electric field, as shown in Fig. 5d, where the data points correspond to SHG (~400 nm) and white-light generation (480 nm) maxima for particular states of input light polarization.

Similar to the previously discussed behavior of the SHG signal, the highest-intensity white light generated (Fig. 5c) was obtained for excitation with a 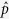-polarized beam. However, contrary to the minimal SHG obtained with the 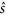-polarized beam, in the case of white light, there was no real minimum for that polarized beam. The local minima of generated light can be observed when the pump polarization is rotated by 45° relative to the 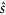
- and 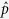
- polarization. This partial following of the SHG polarization behavior by the white-light emission may suggest that observed broad luminescence has other origins besides the phase matching conditions.

**Figure 6:**
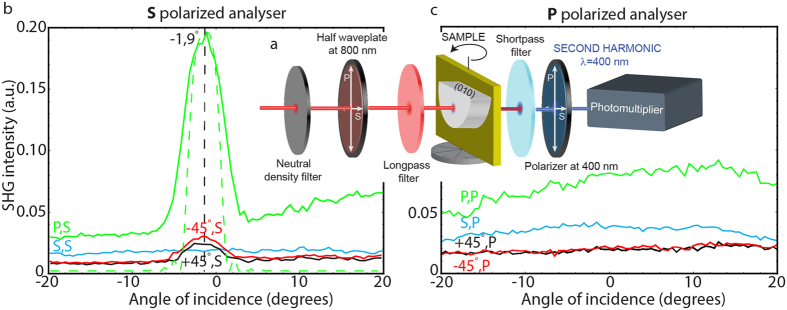
Angular dependence of the nonlinear effects (SHG) in the ZnWO_4_ single crystal. (**a**) Experimental setup used to study the angular dependence showing the rotation stage, photomultiplier and analyzer for angular effects. Angular dependence of SHG for (**b**) the s-polarized outgoing beam, and (**c**) the p-polarized outgoing beam. Experimental data are represented by solid lines and theoretical data by dashed lines.

More details of the polarization dependence of the observed nonlinear effects were obtained by replacing the spectrometer with a photomultiplier and a polarizer (analyzer) before the detector (Fig. 6a). To identify the SHG signal in the broadband light generation, we applied a 40 nm passband filter centered at approximately 400 nm. The sample was placed on a rotating stage and was investigated in the range from −20° to +20°, to study phase-matching condition, with respect to the incident beam (Fig. 6b,c). The analyzer was set up in 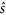
- (Fig. 6b) and 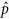
- (Fig. 6c) polarization; the input polarizations used were 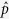, 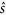 and ±45°.

As expected from the fulfillment of the phase-matching conditions, the highest recorded SHG signal corresponds to 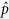-polarized input light (Fig. 6b) and an 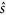-orientation of the analyzer, at almost normal incidence. Indeed the ZnWO_4_ refractive index for the 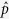-polarized pump at ~800 nm and the refractive index for the 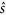-polarized second-harmonic field have similar values (Fig. 4). The maximum SHG was observed for an angle of incidence of –1.9°, which corresponds to a 1.9° cut of the sample towards the (010) crystallographic plane. Thus the highest intensity of SHG by the ZnWO_4_ sample occurs when 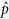-polarized light is parallel to the (010) plane of the crystal and the outgoing light is rotated by 90° relative to the input beam (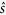 polarization). This is consistent with ZnWO_4_ dispersion relation, and provides the phase matching conditions (Fig. 4).

For the input polarization rotated by ±45° in relation to the 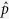-polarization, a small SH peak is also observed at −1.9° and the signal is 10 times less intense in comparison to the SH generated by a 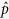-polarized incidence beam. This suggests that PM conditions are not completely fulfilled for this polarization state. For the 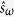 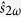 settings of polarization the phase matching conditions are not fulfilled, and a flat signal is recorded. The level of this signal is slightly higher than in the case of the ±45° rotated laser beam, which is caused by the contribution of white-light photons generated by three-photon interactions. For outgoing 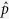-polarized light (Fig. 6c), no SH peak is observed for any polarization of the input beam, and the detected signal is caused by photons of generated white light. A higher signal was registered (Fig. 6c) when the input beam is 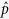-polarized, due to the maximum white-light generation for 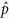 polarization and the higher numbers of photons detected by the detector (Fig. 5c). Summarizing the intense second harmonic generation is possible in ZnWO_4_ only for 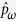 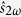 settings of polarization.

**Figure 7:**
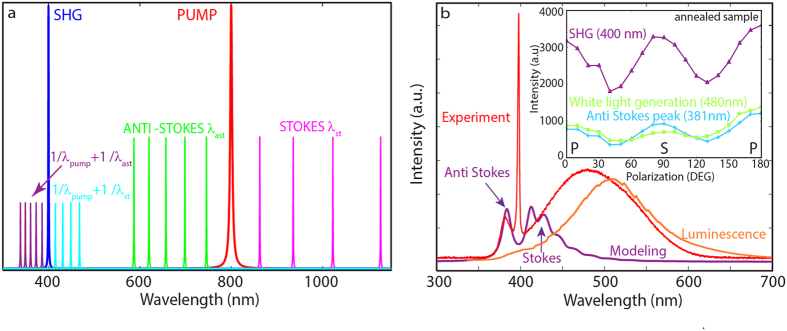
Explanation of the potential causes of the white-light generation in single-crystal ZnWO_4_, such as the Raman-induced sum-frequency generation and luminescence excited with the third harmonic. (**a**) Wavelengths resulting from the sum-frequency generation between the excitation with an 800-nm Ti-sapphire laser and anti-Stokes and Stokes wavelengths. (**b**) Comparison of the experimental spectrum (red) with the fitted Raman anti-Stokes and Stokes contribution (blue) calculated for the first nine components and the UV-excited luminescence (orange). The inset in (**b**) shows the polarization dependence of SHG and white-light generation in annealed samples.

White-light generation is the result of multiphoton processes. One of the contributions is from the three-photon luminescence, which occurs at shorter wavelengths than the observed SHG signal (Fig. 3). Simultaneous three-photon excitation and/or absorption of the generated third-harmonic signal (at 266 nm), above the ZnWO_4_ absorption edge, leads to the material excitation, and consequently white-light photoluminescence. Emission bands in bulk ZnWO_4_ can be attributed to the 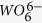 octahedral complex and to a slight deviation from perfect order in the crystal structure, with an excitation peak at 254 nm^30^,^31^, as confirmed by a photoluminescence spectra showing a broadband emission with a peak at 498 nm following UV excitation at 326 nm (Fig. 7b). Previous research has reported the emission peak to be at approximately 480 nm^32^ and the red shift is likely due to the longer laser excitation wavelength.

From Fig. 5, we note that white-light emission is enhanced for the 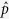-polarized pump –the condition that also maximizes SHG – so white-light must originate from a second-order nonlinear interaction. This effect is related to the cascaded third-harmonic generation resulting from the second-order nonlinear sum frequency generation between the pump field and the generated second-harmonic field, both propagating inside the crystal. With respect to a typical photoluminescence experiment, where the UV field cannot propagate inside the crystal and is fully absorbed within a few microns of penetration depth, cascaded third-harmonic generation is obtained inside the crystal, with the medium transparent to both the pump and SH fields. This process results in an increased interaction volume and a larger effective cross-section for the three-photon luminescence process.

Other higher-order nonlinear effects that contribute to white-light generation partly have their origin in the sum-frequency generation governed by Raman scattering. In particular, ZnWO_4_ shows efficient (>50%) picosecond multiple Stokes and anti-Stokes generation when it is used as a Raman-active crystal in solid-state lasers, based on stimulated Raman scattering (SRS)^33^. In the latter case, the strong SRS-active Raman mode at 907 cm^−1^ is the internal stretching W-O Ag-mode in the 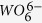 octahedra^17^.

Because of this strong vibrational mode with ωR1 = 907 cm^−1^ in the ZnWO_4_ single crystal, strong anti-Stokes and Stokes components can be excited around the pump wavelength. If these components couple to the strong fundamental pump signal at λ_PUMP_ = 800 nm with the proper phase-matching condition, this process can lead to optical sum-frequency generation, resulting in satellite peaks around the SH. For example, coupling of the first anti-Stokes component at λ_AS_ = 746 nm and the pump results in the signal at λ_BLUE_ = 386 nm, which could explain the unusual peak at 381 nm that appears in the spectrum of the annealed ZnWO_4_ sample, Fig. 7a. The maximum of this peak is blue-shifted from the SHG and it follows the polarization dependence of the SHG signal but with an intensity approximately five times lower, Fig. 7b (inset). This peak appears in the spectra of the annealed sample due to greater crystallinity and correspondingly higher Raman gain.

The modeled Raman spectrum of the ZnWO_4_ single crystal obtained using our laser excitation parameters is given in Fig. 7 (see Supporting Information for model details). The Stokes and anti-Stokes peaks generated by the pump are not observed in Figs 3 and 5b because we applied a shortpass optical (blue) filter during the measurement. In the spectral analysis experiment (Fig. 7b), only the additional peak at 381 nm can be clearly distinguished and it matches the modeled anti-Stokes peak well. The Stokes peaks are probably obscured by other effects, such as the previously mentioned three-photon luminescence in ZnWO_4_.

## Conclusions and Outlook

We reveal that ZnWO_4_ single-crystal has the ability to modulate the frequency of a pump wave via second-harmonic generation. ZnWO_4_ was previously not considered to be a second-harmonic generator because of its centrosymmetric character (P2/c space group). We attribute the origin of the observed SHG signal to multipolar polarization sources, rather than electric dipole polarization sources as in non-centrosymmetric materials, in combination with local symmetry distortion. Phase matching in the ZnWO_4_ crystals, prepared by the micro-pulling-down method, is easily achieved by cutting the samples perpendicular to the growth direction. This results from the natural strong anisotropy of this material in the wavelength range near λ_ω_ = 800 nm→λ_2ω_ = 400 nm. There are a number of other factors that could be explored with a view to improving SHG efficiency, including growth conditions and post-growth processing. Especially important is the development of annealing procedures (atmosphere, temperature and time), high-class polishing and optimizing the thickness of the final crystal sample. The natural phase matching, and low cost of manufacturing of ZnWO_4_ single-crystal make it a good candidate for use in nanoscale imaging of the dynamics of processes such as energy transfer, charge separation, and relaxation paths of excited states. Traditionally pump-probe spectroscopy is used for such studies, however its resolution is limited to macroscopic samples. Recently the idea of combining a broadband femtosecond pump-probe spectroscopy and a confocal microscope was proposed, in which two nonlinear materials produce: (i) first the second-harmonic to generate ultrafast pump pulses at short wavelengths, and (ii) second the white light as a probe^34^. Polli *et al*. demonstrated that such a system could be used to study ultrafast dynamics in polymer blends and organic molecules at the nanoscale. Using ZnWO_4_ in such system as a source of both SH and white-light at the same time would simplify the setup, and potential additional functionalities of the method.

In addition, the ability to use an 800-nm wavelength pump to generate intense white light across almost the entire visible spectrum (380–600 nm) is potentially valuable for a number of applications such as a nonlinear source of white light.

## Experimental

### Crystal growth

Rods of ZnWO_4_ crystals a few centimeters in length and approximately 3 mm in diameter were grown at the Institute of Electronic Materials Technology (ITME) in Warsaw using the micro-pulling-down (μ-PD) method and principle of this idea was described by P. Rudolf, T. Fukuda^35^. High-purity oxide powders of ZnO (99.999%) and WO_3_ (99.99%) produced by Alfa Aesar were used as starting materials. The oxides were mixed with ethanol in an agate mortar and then dried at 350 °C in air in an oven. After drying, the material was placed in the metallic crucible and heated. The material was then transferred to the μ-PD apparatus for crystal growth, where it was seed-grown with a <001> strontium-lanthanum gallate (SrLaGaO_4_) single crystal. Depending on the shape and size of the applied die, the material can be made in the form of a fiber, rod or plate^36^. The μ-PD apparatus, made by Cyberstar, enables the growth of rods up to 45 cm in length. The single crystalline rods were obtained from an iridium crucible in a nitrogen atmosphere with a 0.2 mm/min pulling rate. The use of the crucible with a 0.6-mm-diameter nozzle increased the material volume flowing out of the crucible and increased the speed of wetting of the bottom of the crucible by the outflowing material. In the case of a narrower nozzle (0.4 mm), the amount of the outflow was too small to wet the entire bottom of the die, so growth of a bulk crystal was not possible. To decrease the radial temperature gradient, an active iridium after heater and two insulating zirconium dioxide (ZrO_2_) and aluminum oxide (Al_2_O_3_) ceramics were used. The whole process was controlled manually by changing the pulling rate and output power of the power generator at the initial stage of growth and the growth processes were monitored by a CCD camera. Samples polished on two sides and cut from the original ZnWO_4_ rod were used for optical characterization comparing as-grown crystals and those annealed in an air atmosphere at 1000 °C for 12 h.

### Analysis of the crystal composition and crystallographic orientation

Powder XRD measurements were performed on a Siemens D500 diffractometer equipped with a semiconductor Si:Li detector and a K_α_Cu radiation source. The powder diffraction patterns were measured in θ/2θ scanning mode with a step of 0.02° and counting time of 10 s/step. The experimental data were analyzed by Rietveld refinement using the R. A. Young DBWS-9807 program package^37^. The orientation of the crystal was examined using a four-circle KUMA-diffraction KM4 diffractometer with a K_α_Cu radiation source.

### Linear optical properties

Transmittance measurements were conducted with a CRAIC 20/20 PV microspectrophotometer with Carl Zeiss quartz optical components and integrated imaging system. The light source was a Xe lamp and the detection system was a CCD matrix with 1024 × 58 pixels and spectral resolution of 0.45 nm over a 200–1000 nm spectral range. The photoluminescence characteristics of the material were studied with a Horiba LabRAM system. The sample was excited with a He-Cd laser at 325 nm and emission spectra were collected with a Si CCD detector.

### Nonlinear optical properties

SHG measurements were conducted at the Sapienza University of Rome by means of a collinear scheme working in transmission mode. The input light source was a pulsed Ti:sapphire laser at 795 nm (central wavelength 795 nm, bandwidth 11 nm, pulse duration 130 fs, repetition rate 1 kHz with average power 70 mW). The input beam was 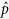-polarized and controlled by a rotating λ/2 plate. The sample was placed on a motorized stage, allowing for variation of the sample rotation angle from –20° to +20°. The output light was filtered by a shortpass filter (the spectrum is presented in Fig. 3) to remove the strong pump light and then analyzed by a fiber spectrometer. To measure the angular dependence, the spectrometer was replaced by a photomultiplier preceded by a polarizer (analyzer) that can be set to either 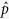
- or 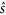-polarization. A 40-nm bandpass filter centered at 400 nm was used to control the spectral selectivity of the photomultiplier.

## Additional Information

**How to cite this article**: Osewski, P. *et al*. Self-Phase-Matched Second-Harmonic and White-Light Generation in a Biaxial Zinc Tungstate Single Crystal. *Sci. Rep.* **7**, 45247; doi: 10.1038/srep45247 (2017).

**Publisher's note:** Springer Nature remains neutral with regard to jurisdictional claims in published maps and institutional affiliations.

## Supplementary Material

Supplementary Information

## Figures and Tables

**Figure 1:**
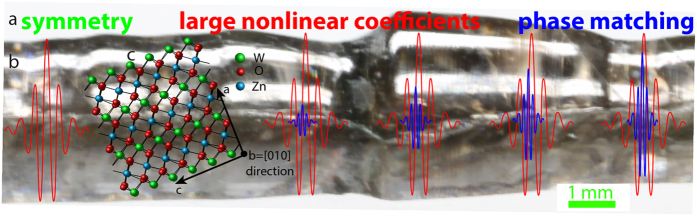
Second-harmonic generation in ZnWO_4_ crystal. (**a**) Optical parameters required to realize SHG are non-centrosymmetric character of the medium, phase-matching conditions enabled by medium birefringence, and large nonlinear optical coefficients; (**b**) ZnWO_4_ crystal grown by the micro-pulling-down method; (**c**) Orientation of the pump wave towards the [010] axis of ZnWO_4_, which enables the phase-matching conditions and thus second-harmonic generation.
