# Supplementary material for: Self-Phase-Matched Second-Harmonic and White-Light Generation in a Biaxial Zinc Tungstate Single Crystal
Source: Sci Rep. 2017 Mar 24;7:45247. doi: 10.1038/srep45247 (PMC5364410; doi:10.1038/srep45247)
Supplement: Supplementary Information [file srep45247-s1.pdf]

Supporting Information

Self-Phase-Matched Second-Harmonic and White-Light Generation in a Biaxial Zinc Tungstate Single Crystal

Pawel Osewski, Alessandro Belardini, Emilija Petronijevic, Marco Centini, Grigore Leahu, Ryszard Diduszko, Dorota Anna Pawlak, and Concita Sibilia

Like other sheelites and wolframites of the form BWO_4 (B = Ca, Sr, Ba, Sb), $ZnWO_4$ is known for its efficient SRS properties when used as a Raman-active crystal in solid-state lasers or as a frequency converter.[16,21] The strongest Raman mode at 907 cm^{-1} is due to the internal stretching of the W-O A_g mode of the WO_6 octahedra, which leads to multiple Stokes and anti-Stokes generation with efficiencies up to 50%.[16,20] We thus expect that in the annealed sample, there could be efficient multiple Raman generation induced by the pump field. Anti-Stokes (Stokes) signals can further couple with the fundamental beam and through a proper phase-matching-governed sum-frequency generation (SFG), they can generate fields at shorter (longer) wavelengths with respect to the SH peak.

Analyzing Figure 3, we note that the annealed sample shows a peculiar peak close to the SHG peak, centered at 383 nm, which is approximately the wavelength resulting from the SFG between the first anti-Stokes line (at 746 nm) and the fundamental field (at 795 nm). To provide a tentative fit with the experimental data, we modeled the SFG process by solving the system of three coupled equations:

$$\frac{dA_1}{dz} = \frac{8\pi i d\omega_1^2}{k_1 c^2} \cdot A_3 \cdot A_2^* \cdot e^{-i\Delta kz},$$
$$\frac{dA_2}{dz} = \frac{8\pi i d\omega_2^2}{k_2 c^2} \cdot A_3 \cdot A_1^* \cdot e^{-i\Delta kz},$$
$$\frac{dA_3}{dz} = \frac{8\pi i d\omega_3^2}{k_3 c^2} \cdot A_1 \cdot A_2 \cdot e^{i\Delta kz},$$

where A_i, ω_i and k_i (i=1,2,3) are the field's amplitude, the angular frequency and the wave vector, respectively, $d = \chi^2/2$ is the second-order effective nonlinear optical susceptibility and Δk is the phase mismatch:

$$\Delta k = k_1 + k_2 - k_3.$$

We attribute the origin of the second-order nonlinear optical susceptibility to a multipolar source terms of the nonlinear polarization.[24]

In our case, (k_1, A_1) corresponds to either the anti-Stokes or Stokes generation, (k_2, A_2) corresponds to our fundamental beam and (k_3, A_3) stands for the generated sum-frequency component. It is natural to assume that the processes are taking place under the undepleted pump approximation; thus $A_2(z) = A_2(0)$. Because this is a spontaneous Raman scattering process, we assume that the intensity of the Raman signal is much lower than the pump, which gives us the intensity of the SFG leaving the crystal (z=L):

$$I_3 \approx \frac{256\pi^4 d^2}{n_3^2 \lambda_3^2} \cdot I_1 \cdot I_2 \cdot L^2 \cdot sinc\left(\frac{\Delta k \cdot L}{2}\right)^2,$$

$I_i = 2n_i\varepsilon_0 c|A_i|^2$ (i=1,2,3).

Both the SHG and the sum-frequency generation between the pump and the Raman signals are driven by the same second-order nonlinear response tensor; thus they should effectively have the same polarization dependence. In the inset of Figure 7a, the peak near 383 nm follows the polarization dependence of the SHG signal, which proves our assumption that this peak is due to the coupling between the pump and the first anti-Stokes signal.

However, from the measurements reported in Figure 3**,** there is no direct evidence of Raman spectra around the pump wavelength because we used a blue filter (with a 620-nm cutoff) to prevent detector saturation from the strong pump signal. For this reason, we have separately measured the Raman shift spectra; our results (Figure S1) are in good agreement with those previously reported in the literature.[16,20] In particular, we observe the strong vibrational mode at $\omega_{mode} \approx 907cm^{-1}$ with bandwidth ~15 cm^{-1}. Anti-Stokes and Stokes generation peaks are observable at the wavelengths given by the following relations:

$$\lambda_{AntiStokes}(= \lambda_1) = \frac{1}{\frac{1}{\lambda_2} + \omega_{mode\_n} \cdot 10^{-7}},$$

$$\lambda_{Stokes}(= \lambda_1) = \frac{1}{\frac{1}{\lambda_2} - \omega_{mode\_n} \cdot 10^{-7}},$$

where λ_2 is the pump wavelength and $\omega_{mode\_n} = n \cdot \omega_{mode}$ is the n-th order of the mode. In principle, these modes can further couple with the pump. However, the efficiency of the SFG process is governed by the phase-matching condition (which is fulfilled only around the SH wavelength) and limited by the decreasing intensity I_1 of the Raman peaks when higher-order modes are considered.[20] These aspects have been taken into account in our calculations (Figure 7b) revealing that only the lower-order modes can significantly contribute to the generated spectrum around the SHG wavelength. In particular, the strong evidence of the peak at 383 nm is related to the best fulfillment of the phase-matching condition between the pump and the first anti-Stokes line. We cannot clearly distinguish the peaks related to SFG between the pump and Stokes lines because they are probably covered by other broadband competing processes (in other words, the previously mentioned three-photon luminescence).

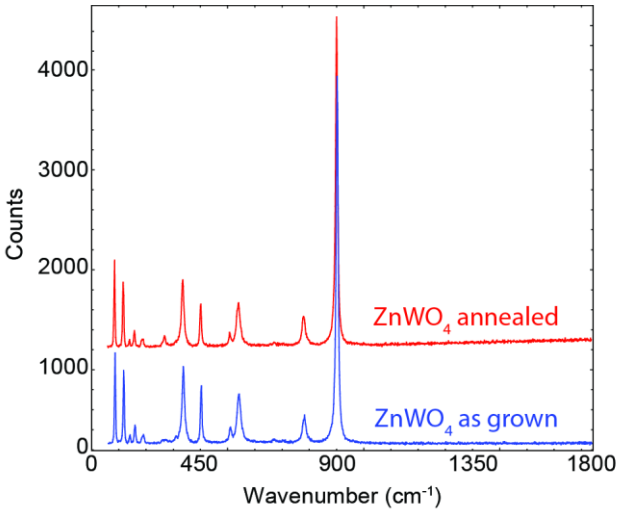

Figure S1. Raman spectra of the as-grown and annealed $ZnWO_4$ single crystals.
